# A Structurally Diverse Heterocyclic Library by Decoration of Oxcarbazepine Scaffold

**DOI:** 10.3390/molecules181113705

**Published:** 2013-11-06

**Authors:** Luca Vaghi, Emanuela Calcio Gaudino, Giancarlo Cravotto, Giovanni Palmisano, Andrea Penoni

**Affiliations:** 1 Dipartimento di Scienza e Alta Tecnologia, Università degli Studi dell' Insubria, via Valleggio 11, Como 22038, Italy; E-Mails: giovanni.palmisano@uninsubria.it (G.P.); andrea.penoni@uninsubria.it (A.P.); 2 Dipartimento di Scienza e Tecnologia del Farmaco, Università degli Studi di Torino, via Giuria 9, Torino 10125, Italy; E-Mails: emanuela.calcio@unito.it (E.C.G.); giancarlo.cravotto@unito.it (G.C.)

**Keywords:** oxcarbazepine, heteroannulation, library of compound, fused heterocycles, cyclization, cyclocondensation

## Abstract

A library of new heterocyclic systems was synthesized starting from oxcarbazepine (OXC, Trileptal^®^, 10-oxo-10,11-dihydro-5*H*-dibenzo[*b,f*]azepine-5-carboxamide). The key for these transformations is the α-enolizable ketone present on the [*d*]-side of our starting material OXC, thus, an in depth investigation of the literature to find heteroannulation reactions for substrates carrying an α-enolizable ketone gave us a boost to discover an excellent derivatization strategy and [3+2], [4+2] and [4+1] approaches were successfully developed. Almost always a pre-functionalization was needed, but also the direct one-pot heterocycle construction was also explored.

## 1. Introduction

**Figure 1:**
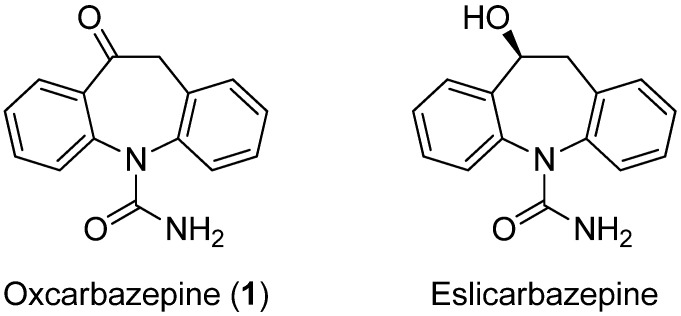
Structures of oxcarbazepine and eslicarbazepine.

Oxcarbazepine (OXC, Trileptal^®^, 10-oxo-10,11-dihydro-5*H*-dibenzo[*b,f*]azepine-5-carboxamide, **1**, Figure 1), known since 1965 [1] and patented by Ciba-Geigy [2] represents the first narrow-spectrum antiepileptic drug (AED) in several years to be FDA-approved in 2000 as a monotherapy for the treatment of partial seizures in adults [3,4].

Although the precise mechanism by which OXC exerts its antiseizure effect is unknown, it is well documented that OXC is completely adsorbed and extensively metabolized (approximately 70%) by reductase enzymes to its pharmacologically active 10-monohydroxy derivative form eslicarbazepine ((*S*)-licarbazepine, MHD, Figure 1) [5].

The structure-activity relationships to date have shown that the potency and selectivity of **1** are linked to the *N*-carboxamido group embodied in the twisted boat conformation of the dibenzoazepine core [6]. Accordingly, subsequent synthetic efforts focused on chemical manipulation of the 10-position, mimicking the active MDH with the aim of improving the pharmacological profile [7,8]. At present, an in-depth manipulation of **1** in order to find activities other than the anticonvulsant one has not been previously undertaken. Due to our interest in heterocyclic chemistry [9,10,11,12,13,14] and to the availability in large quantities of **1**, we envisioned that **1** would be a versatile key intermediate for further decoration of the azepine subunit, to obtain a new library of compounds of high potential value in the drug research field. We report herein experimental procedures for the synthesis of compounds based on the template **1** (a large part of them with intact *N*-carboxamido functions). Most reactions were run only once and the reported yields are therefore unoptimized. A diverse set of 5- and 6-membered heterocyclic rings (e.g., oxazole, pyrrole, indole, thiazole, pyrazine, quinoxaline etc) were appended, thereby paving the way to [*d*]-fused heterocycles which have been largely neglected or are totally absent in the literature. This library of compounds will be subjected to high throughput screening for deciphering their biological activity and these results will be reported in due course.

## 2. Results and Discussion

**Scheme 1:**
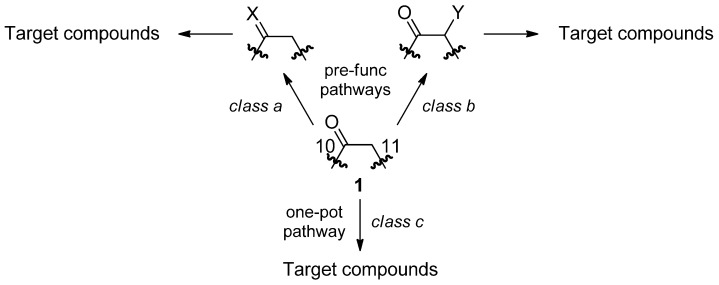
Synthetic strategy *en route* to new fused heterocycles from oxcarbazepine (**1**).

The *de novo* synthesis of heterocyclic ring systems represents one of the most important topics and this goal was frequently achieved by cyclization strategies. Capitalizing on the presence of a carbonyl function on the [*d*]-side of OXC, the molecules described in this work could be structurally categorized in three classes depending on the type of pre-functionalization of **1** required prior to the heterocyclization step: pre-functionalization of position 10 (*class a*); pre-functionalization of position 11 (*class b*); no pre-functionalization required (*class c*) (Scheme 1).

### 2.1. Class a

**Scheme 2:**
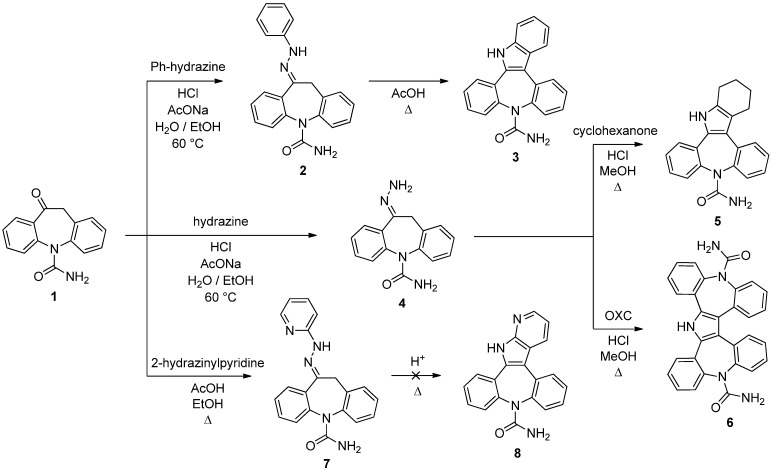
[3+2] Fischer indolization like strategy.

The venerable (first reported over 120 years ago) Fischer reaction still stands out as one of the most efficient approach to indoles [15,16]. A number of literature methods for indolization of enolizable carbonyls with aryl hydrazines were examined in an effort to convert **1** to **3** (Scheme 2). Attempts to carry out this transformation (with phenylhydrazine and **1** as [C-C-N] and [C-C] units, respectively, in a [3+2] strategy) in a one-pot procedure under a variety of solvents and acid catalysts resulted in formation of dark mixtures in which only trace amounts of **3** could be detected.

Considerably better results were obtained starting from the known 10-phenylhydrazone **2** [8] which, on exposure to refluxing acetic acid for 1 hour, underwent the desired Fischer indolization and **3** was isolated in 79% yield. However, much to our surprise, exposure of pyrid-2-yl hydrazone **7** to the same reaction conditions did not afford the anticipated product **8** but rather yielded an intractable mixture of unidentified products. This reaction was attempted several times, producing, in all cases, similar results.

We planned to apply the Fischer strategy using the 10-hydrazone **4** [8] as [C-C-N] unit and cyclohexanone and OXC (**1**) itself as [C-C] units. In this context we found the hydrazone formation/Fischer indolization (MeOH, HCl, 68 °C, 30 min.) was the most effective route and therefore **5** and **6** were prepared without significant complications in 69% and 58% yield, respectively. Compound **6** is formally the product of a Piloty-Robinson reaction on the transient azine [17,18].

**Scheme 3:**
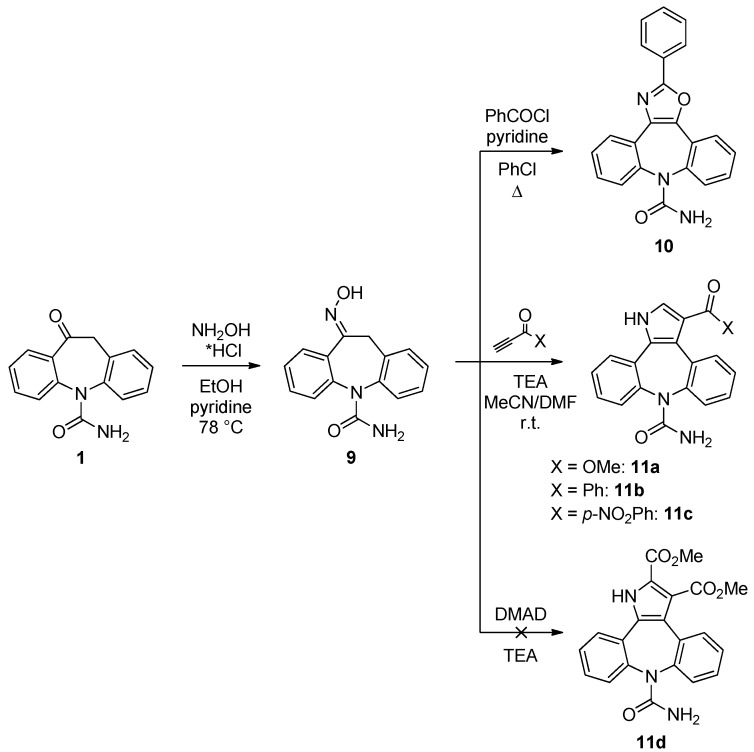
[3+2] Approach with oxime **9** as [C-C-N] unit.

The use of oxime **9** [8] as [C-C-N] unit in a [3+2] approach facilitated considerably the preparation of oxazole- and pyrrole-fused analogues (Scheme 3). In the event, cyclocondensation of **9** with benzoyl chloride in the presence of pyridine (PhCl, 132 °C, 30 min.) [19] gave oxazole **10** in fair (40%) yield. Furthermore, **9** is ideally suited to serve as a starting material to prepare pyrrolo[2,3-*d*]azepines **11** by using the modified methodology of Trofimov [20]. Accordingly, **11a** was obtained by reaction of **11** with methyl propiolate catalysed by TEA in MeCN-DMF (4:1) at r.t. for 1 hour. The transient *O*-vinyl oxime derivative underwent a tandem [3,3]-sigmatropic rearrangement‒ cyclocondensation to **11a** (57% isolated yield). This reaction is usually reported [21] to require more drastic conditions (DMSO, >90 °C) than those reported here. It was found that this approach could be successfully extended to other electron-deficient alkynes (e.g., 1-phenyl-2-propyn-1-one and its 4-nitrophenyl derivative) leading to **11b** and **11c** in 71 and 78% yield, respectively. It is worth mentioning that reaction of more electrophilic DMAD with **9** under the same conditions failed to provide the expected pyrrole derivative **11d**, a consequence presumably of the overwhelming steric effect of the additional CO_2_Me in the six-membered cyclic transition state.

**Scheme 4:**
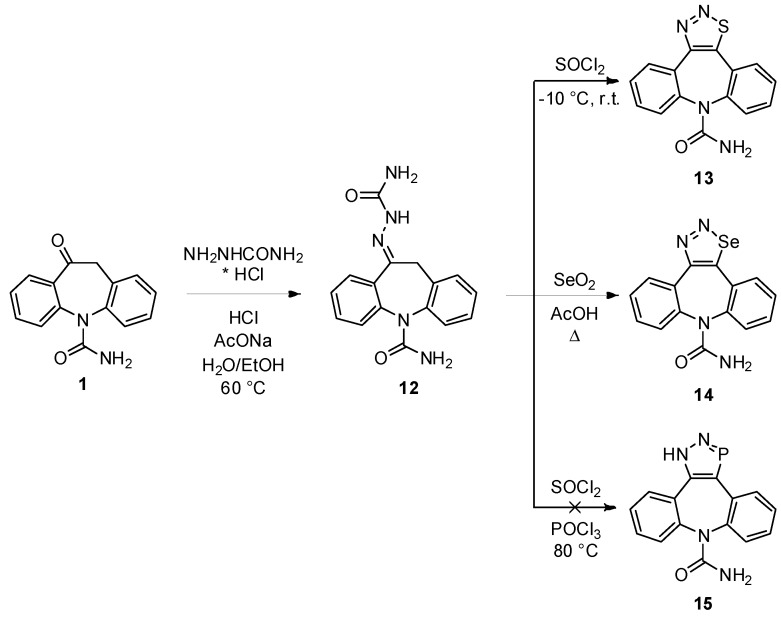
[4+1] Approach to five-membered diaza heterocycles.

The [4+1] approach is rarely used in the annulation of a carbocyclic scaffold, but it represents the basic mode of accessing heterocycles embodying a N-N-X subunit (Scheme 4). To this end, hetero-cyclization of semicarbazone **12** (as [C-C-N-N] unit) [8] to the corresponding 1,2,3-thiadiazole **9** (83%) was readily accomplished with SOCl_2_ ([S] unit) [22]. By analogy to the above achieved Hurd-Mori reaction, the treatment of **4** with SeO_2_ [23] in refluxing AcOH also proceeded to furnish primarily the selenadiazole **10** in 48% yield. A 2007 report by Huazhou *et al.* [24] describes the high-yielding generation of 2*H*-1,2,3-diazaphosphole (starting from semicarbazones) through the agency of a POCl_3_-SOCl_2_ mixture and subsequent treatment with EtOH. However, exposure of **4** to these conditions failed to provide the corresponding diazaphosphole **15** and no further time was invested to understand this failure.

### 2.2. Class b

**Figure 2:**
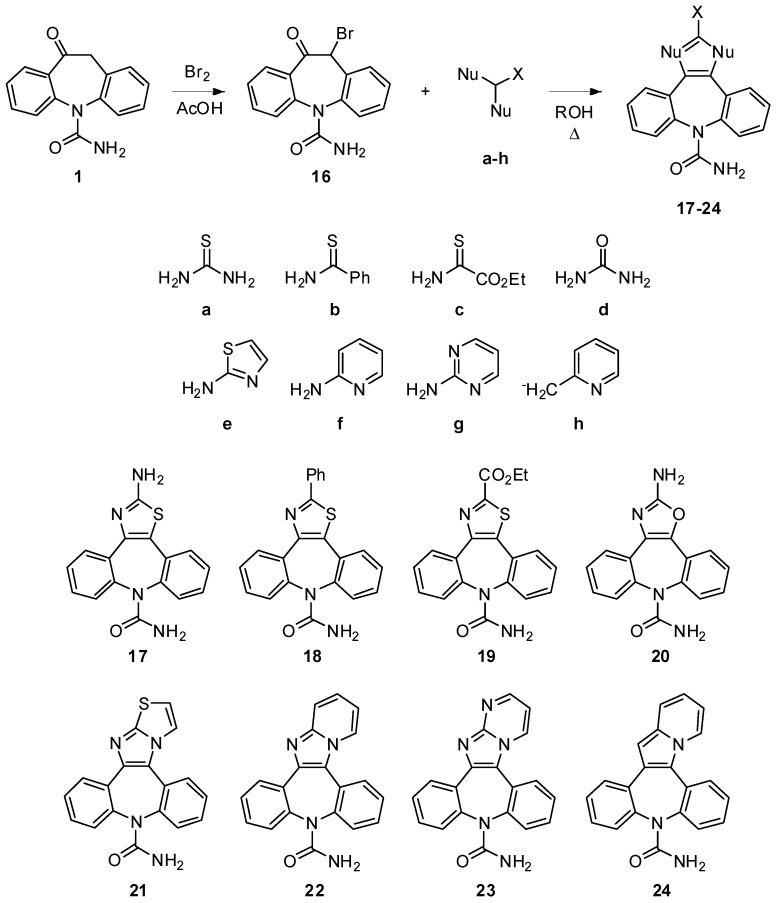
Reaction of the 11-bromo derivative (**16**) with 1,3-bis(nucleophiles).

In the second approach (pre-functionalization of the position 11) α-bromo ketone **15** served as pivotal intermediate for further elaboration. It is well known that α-halocarbonyls have been successfully exploited in the building of numerous 5- and 6-membered heterocycles [25]. The bromo ketone **16** was prepared by a literature procedure [26] and its reaction with thiourea (Hantzsch reaction) in refluxing EtOH proceeded smoothly and consistently to provide the thiazole derivative **17** in a pleasing 43% yield. Spurred by this finding, we set out to explore the reactions of **15** with other 1,3 *N,S-*bis(nucleophiles) (*i.e.*, benzothioamide **b** and ethyl 2-amino-2-thioxoacetate **c**) (Figure 2).

Surprisingly, it was not possible to prepare the corresponding thiazoles **18** and **19**. Reaction conditions (refluxing EtOH) similar to those for **17** gave no reaction, while higher temperatures (up to 120 °C in a heavy wall-pressure tube) or prolonged reaction times (up to 6 days) gave inconsistent or messy results. It seemed likely that this failure was due to product/reagent decomposition which ensued at the high temperatures and extended reaction times necessary to heterocyclization, so more robust reagents might be desirable. Accordingly, we chose to examine a structurally diverse set of 1,3 *N*,*N*- (*i.e*., 2-amino thiazole **e**, 2-aminopyridine **f** and 2-aminopyrimidine **g**), 1,3 *N,O*- (urea **d**) and 1,3 *C*,*N*- bis(nucleophiles): (2‑methylpyridine **h**) (Figure 2). These substrates were expected to be even less reactive, but these difficulties have been circumvented by switching to butan-1-ol as a solvent with ideal boiling point, solubilization characteristics, and polarity.

We found that heating **16** with 2-aminothiazole **e** in refluxing *n*-BuOH for 6 h did indeed effect cyclization, providing primarily the thiazole **21** as the only identifiable product in 56% yield. The optimized reaction conditions were applied to the other representative *N,N*-, *N,O-* and *C,N*-bis(nucleophiles). Generally, the yields are satisfactory (32%–53% range), the exception being 2-methylpyridine **h**, which did not perform well in the Tschitschibabin indolizine synthesis (*viz*. condensation of α-haloketones with 2-alkylpyridines) [27]. The fact that **h** did not give any noticeable quantity of the expected **25** is rather difficult to interpret, and we cannot explain this discrepancy at the moment. Interestingly, routes a and b provided access to an array of diversely substituted derivatives with the *N*-carboxamido group still attached to azepine core.

**Scheme 5:**
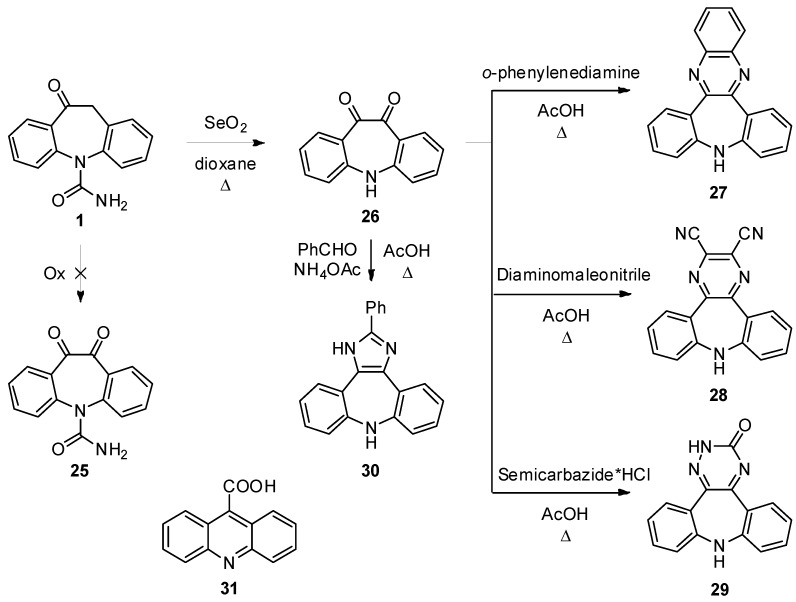
Products of the cyclocondensations on diketo compound **26**.

Another approach to compounds belonging to class b entailed the installation of an α-dicarbonyl function at the [*d*]-edge of the azepine subunit in **1** (to **25**) followed by the heterocyclization step (Scheme 5). The two-fold benzylic oxidation of carbamazepine **CBZ** in the presence of a metal salt and *N*-hydroxyphthalimide has been shown by Alsters *et al* [28] to give the required **25** (in only 8% at best!) and, as far as we are aware, this constitutes the only synthesis of this compound. Owing to the availability in large quantities of **1**, efforts were taken to oxidize it using the Alsters conditions. However, it appeared that this methodology is of limited applicability even to our case. Different methods for oxidation of enolizable carbonyl to α-diketones were tested, but they uniformly failed under a range of conditions: the starting material either proved inert or underwent a messy reaction. On exposure to freshly sublimed SeO_2_ in refluxing dioxane (7 h) the desired **25** was not observed. Instead, α-diketone **26** was produced as the single reaction product, which crystallized from the crude reaction mixture in 90% yield. OXC reacted also with benzeneseleninic anhydride in chlorobenzene at 120 °C for 6 h to provide the same diketone (87%). Unfortunately, despite considerable efforts, the oxidation of **1** without compromising the pendant *N*-carboxamido group proved to be fruitless and it was therefore decided to pursue this approach using **26** as a starting material. Actually, reaction of derivatives devoid of *N-*carboxamido function with chlorosulphonyl isocyanate (CSI) would help address this problem [29].

1,2-Dicarbonyl compounds are versatile intermediates for further elaboration leading*, i.e*., to five- to seven-membered ring systems and **26** proved to be no exception. Specifically, treatment of a solution of **26** in AcOH with *o*-phenylenediamine (118 °C, 1 h) effected conversion to the quinoxaline derivative **27** [30].

Following this procedure, either diaminomaleonitrile or semicarbazide hydrochloride participated as 1,4 *N,N*-bis(nucleophiles) leading to the isolation of pyrazine **28** and triazinone **29** in 65 and 19% yield, respectively. Further structural diversity could be achieved via MultiComponent Reactions (MCRs). In this context, the reaction of 1,2-dicarbonyls with an aldehyde and NH_3_, originally developed by Debus and Radziszewski [31], is a remarkably effective method for the preparation of 1*H*-2,4,5-trisubstituted imidazoles. Thus, the 1*H*- imidazole **30** could be accessed, albeit in a modest 28% yield, from reaction of **26** with benzaldehyde and NH_4_OAc (as a source of NH_3_) in AcOH at 80 °C.

The disappointing yields obtained by reacting **26** under acidic conditions represent the manifestation of the vexing problem associated with ring contraction-dehydration process leading to **31** as an end-point product.

### 2.3. Class c

**Scheme 6:**
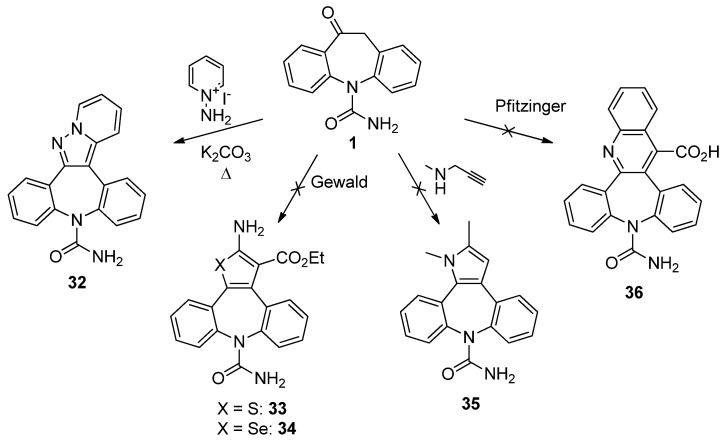
Attempts to direct heteroannulation.

In principle, OXC *per se* could also be the source of additional analogues (Scheme 6) without the need of pre-functionalization (class c). Thus, access to the pentacyclic pyrazolo[1,5-*a*]pyridine core in **32** (22%) was easily gained via thermal [3+2] assembly by base-promoted condensation (H_2_O/*i*-PrOH 1:1, K_2_CO_3_, 100 °C, 2 h) of 1-aminopyridinium iodide (as [N-N-C] unit) and **1**.

Nevertheless, the participation of **1** in other cyclocondensation reactions proved to be a tricky undertaking. The application of Gewald conditions [32] to the reaction of **1** with ethyl cyanoacetate and sulphur in the presence of diethylamine (MeOH, 65 °C, 20 h) provided only extensive decomposition products and no isolable quantities of the expected 2-aminotiophene derivative **33**. Furthermore, attempts at replacing sulphur by selenium under the same conditions to achieve a seleno-Gewald multicomponent reaction [33] also gave a complex mixture of products, none of which was the desired 2-aminoselenophene **34**.

In a similar vein, when **1** was subjected to the conditions under which other cyclic ketones were successfully cyclized (*N*-methyl-*N*-propargylamine, PhMe, 110 °C, sealed tube) to annulated [*b*]pyrroles through the intermediacy of a *N*-vinyl-*N*-propargylamine [34], no traces of **35** were detected in the reaction mixture over a 8-day time period. Finally, the reluctance of **1** to participate in these reactions was also witnessed by the failure met in the Pfitzinger protocol (isatin, KOH, H_2_O, 100 °C) [35], so this strategy was not pursued further.

## 3. Experimental

All reactions were performed using standard glassware and IKA num heating plates. Reactions utilizing air-sensitive reagents were performed in dried glassware under a nitrogen atmosphere. Solvents were used as received without further purifications. All reagents, if not otherwise specified, were used as received and, if necessary, stored under inert gas. Oxcarbazepine was supplied by Trifarma SpA, Ceriano Laghetto, Italy. For thin-layer chromatography (TLC) analysis Macherey-Nagel Polygram^®^ sil G/UV 254 pre-coated plates were used. Column chromatography was performed on silica gel 60A (70–200 µm) (Carlo Erba Reagents, Arese, Italy). Melting point (mp) determinations were performed by using a Gallenkamp melting point apparatus (Weiss-Gallenkamp, Loughborough, UK). ^1^H-NMR (400 MHz) and ^13^C-NMR (100 MHz) were measured on a AV400 spectrometer (Bruker Corporation, Billerica, MA, USA). Chemical shifts (δ) are expressed in parts per million (ppm) and coupling constants are given in hertz (Hz). Splitting patterns are indicated as follows: s = singlet, d = doublet, t = triplet, q = quartet, m = multiplet, br = broad, dd = doublet-doublet, td = triplet-doublet. Chemical ionization mass spectra (+ve mode) (CI^+^-MS) were performed on a Finnigan-MAT TSQ70 instrument with isobutane as the reactant gas. Elemental analyses were performed on a Perkin Elmer Series II CHNS/O Analyzer 2400.

*10-(Phenylhydrazono)-10,11-dihydro-5H-dibenzo[b,f]azepine-5-carboxamide* (**2**). The compound was prepared following the procedure present in the literature and the spectroscopic data were in accordance with those reported [8]. Yield: 88%.

*Dibenzo[2,3:6,7]azepino[4,5‑b]indole‑9(14H)-carboxamide* (**3**). A well stirred solution of **2** (410 mg, 1.20 mmol) in acetic acid (5 mL) was brought to reflux for 1 h. The reaction progress was monitored by TLC analysis (SiO_2_, AcOEt, *R_f_* **2** = 0.39, *R_f_* **3** = 0.25). The mixture was then cooled to room temperature and added dropwise to a stirred ice/water solution (150 mL). The precipitate was filtered, washed with water and dried under vacuum to afford **3** as green solid (309 mg, 79%): ^1^H-NMR (DMSO-*d_6_*) δ 11.65 (s, 1H), 7.94 (m, 2H), 7.80 (m, 1H), 7.52 (m, 3H), 7.41 (m, 3H), 7.34 (m, 1H), 7.21 (t, *J* = 7.3 Hz, 1H), 7.14 (t, *J* = 7.5 Hz, 1H), 5.20 (br. s, 2H). ^13^C-NMR (DMSO-*d_6_*) δ 157.2, 141.3, 140.0, 137.6, 135.0, 134.2, 130.9, 130.1, 129.8, 129.6, 128.7, 128.6, 128.2, 128.1, 127.5, 126.4, 123.4, 120.8, 120.1, 113.1, 112.6; m.p. 216–219 °C dec.; MS (CI^+^): *m/z* = 326 [MH]^+^, 283 [(M-HNCO)H]^+^; Anal. Calcd. for C_21_H_15_N_3_O: C, 77.52; H, 4.65; N, 12.91; Found: C, 77.31; H, 4.87; N, 13.01.

*10-Hydrazinylidene-10,11-dihydro-5H-dibenzo[b,f]azepine-5-carboxamide* (**4**). The compound was prepared following the procedure present in the literature and the spectroscopic data were in accordance with those reported [8]. Yield: 45%.

*2,3,4,14-Tetrahydrodibenzo[2,3:6,7]azepino[4,5b]indole-9(1H)-carboxamide* (**5**). Compound **4** (100 mg, 0.38 mmol) and cyclohexanone (45 µL, 0.44 mmol) were dissolved in methanol (15 mL). 37% aq. HCl (47 µL, 0.21 mmol) was added and the reaction mixture was heated at 68 °C for 30 min under stirring. The reaction progress was monitored by TLC analysis (SiO_2_, AcOEt, *R_f_* **4** = 0.04, *R_f_* **5** = 0.22). After cooling to room temperature and removing part of the solvent under reduced pressure, the solution was dropped into stirred ice/water (50 mL). The precipitate was filtered, washed with water and dried under vacuum to gave **5** as yellow solid (85 mg, 69%): ^1^H-NMR (DMSO-*d_6_*) δ 11.20 (s, 1H), 7.55–7.28 (m, 8H), 5.43 (br. S, 2H), 2.90 (m, 1H), 2.72–2.50 (m, 3H), 1.97 (m, 2H), 1.75 (m, 1H), 1.60 (m, 1H); ^13^C-NMR (DMSO-*d_6_*) δ 157.1, 139.3, 139.2, 134.8, 131.8, 130.3, 130.2, 130.1, 128.1, 128.1, 128.0, 127.7, 127.3, 126.9, 126.5, 119.1, 115.8, 24.5, 24.1, 23.5, 23.4; m.p. 268–269 °C; MS (CI^+^): *m/z* = 330 [MH]^+^, 287 [(M-HNCO)H]^+^; Anal. Calcd. for C_21_H_19_N_3_O: C, 76.57; H, 5.81; N, 12.76; O, 4.86; Found: C, 76.38; H, 5.93; N, 12.49.

*5H-Tetrabenzo[b,b',f,f']pyrrolo[2,3-d:4,5‑d']bisazepine-5,14(19H)-dicarboxamide* (**6**). Compound **1** (111 mg, 0.44 mmol) and **4** (117 mg, 0.44 mmol) were dissolved in hot methanol (15 mL) and the mixture was stirred at 68 °C for 10 min. 37% aq. HCl (47 µL, 0.21 mmol) was then added and the solution was left at reflux under stirring for 2 h. The reaction progress was monitored by TLC analysis (SiO_2_, AcOEt/MeOH 95:5, *R_f_* **4** = 0.12, *R_f_* **1** = 0.37, *R_f_* **6** = 0.11). After 30 min the product started to precipitate as white solid. When the reaction was complete the mixture was cooled to r.t., **6** was recovered in a pure form by filtration and dried in vacuum (124 mg, 58%): ^1^H-NMR (DMSO-*d_6_*) δ 12.18 (s, 1H), 7.86 (m, 2H), 7.59–7.53 (m, 4H), 7.45 (m, 4H), 7.34 (m, 4H), 7.15 (m, 2H), 5.77 (br. s, 4H); ^13^C-NMR (DMSO-*d_6_*) δ 157.2, 141.1, 140.1, 134.0, 131.6, 130.8, 130.3, 130.0, 129.9, 129.1, 128.2, 128.1, 128.0, 127.9, 119.5; m.p. 336–337 °C; MS (CI^+^): *m/z* = 484 [MH]^+^, 441 [(M-HNCO)H]^+^; Anal. Calcd. for C_30_H_21_N_5_O_2_: C, 74.52; H, 4.38; N, 14.48; Found: C, 74.69; H, 4.27; N, 14.37.

*10-(2-Pyridinylhydrazono)-10,11-dihydro-5H-dibenzo[b,f]azepine-5-carboxamide* (**7**). A mixture of **1** (500 mg, 2.0 mmol) and 2-hydrazinopyridine (654 mg, 6.0 mmol) in ethanol (20 mL) and three drops of acetic acid was heated at 78 °C for 1 h under stirring. The reaction progress was monitored by TLC analysis (SiO_2_, AcOEt, *R_f_* **1** = 0.26, *R_f_* **7** = 0.07). After allowing to cool to room temperature, the precipitate was filtered, washed with ethanol and dried in vacuum to give (**7**) as yellow solid (410 mg, 60%): ^1^H-NMR (DMSO-*d_6_*) δ 10.24 (br. s, 1H), 8.20 (d, *J* = 4.7 Hz, 1H), 7.95 (m, 1H), 7.69 (m, 1H), 7.43–7.24 (m, 8H), 6.85 (m, 1H), 5.96 (br. s, 2H), 4.32 (d, *J* = 17.6 Hz, 1H), 3.94 (d, *J* = 17.6 Hz, 1H); ^13^C-NMR (DMSO-*d_6_*) δ 158.7, 156.9, 148.5, 144.1, 142.5, 141.8, 138.9, 135.5, 134.6, 130.8, 130.0, 129.7, 129.0, 128.9, 128.8, 128.7, 128.0, 116.6, 108.1, 33.9; m.p. 230–231 °C; MS (CI^+^): *m/z* = 327 [MH]^+^; Anal. Calcd. for C_19_H_15_N_3_O_3_: C, 69.96; H, 4.99; N, 20.40; Found: C, 69.75; H, 5.13; N 20.29.

*10-(Hydroxyimino)-10,11-dihydro-5H-dibenzo[b,f]azepine-5-carboxamide* (**9**). The compound was prepared following the procedure present in the literature and the spectroscopic data were in accordance with those reported [8]. Yield = 94%.

*2-Phenyl-8H-[1,3]oxazolo[4,5-d]dibenzo[b,f]azepine-8-carboxamide* (**10**). **9** (100 mg, 0.37 mmol), pyridine (30 μL, 0.37 mmol) and benzoyl chloride (108 μL, 0.93 mmol) were added to chlorobenzene (5 mL). The mixture was stirred for 30 min at r.t., then the temperature was rise to 132 °C and the reaction was stirred for 30 min. The reaction progress was monitored by TLC analysis (SiO_2_, CH_2_Cl_2_/EtOH 95:5, *R_f_* **9** = 0.13, *R_f_* **10** = 0.22). After cooling to r.t. the solvent was removed under reduced pressure, water (20 mL) was added to the residue and the aqueous phase was extracted with AcOEt (3 × 15 mL). The combined organic layers were washed with H_2_O (2 × 20 mL), dried (Na_2_SO_4_) and the solvent removed under vacuum. Flash column chromatography (SiO_2_, CH_2_Cl_2_/EtOH 95/5, *R_f_* = 0.22) gave **10** as white solid (52 mg, 40%): ^1^H-NMR (CDCl_3_) δ 8.27–8.24 (m, 2H), 8.18–8.16 (m, 1H), 7.88 (dd, *J* = 7.6, 1.5 Hz, 1H), 7.67–7.50 (m, 9H), 4.61 (br. s, 2H); ^13^C-NMR (CDCl_3_) δ 161.7, 157.7, 137.7, 137.6, 137.1, 131.3, 130.8, 130.5, 130.4, 130.3, 130.3, 129.5, 129.3, 129.1, 128.7, 128.7, 127.8, 127.4, 127.2, 125.8; m.p. 230–232 °C; MS (CI^+^): *m/z* = 354 [MH]^+^, 382 [M+C_2_H_5_]^+^, 337 [(M-NH_2_)H]^+^, 310 [(M-HNCO)H]^+^; Anal. Calcd. for C_22_H_15_N_3_O_2_: C, 74.78; H, 4.28; N, 11.89; Found: C, 74.85; H, 4.22; N, 11.79.

*Methyl-8-carbamoyl-1,8-dihydropyrrolo[2,3-d]dibenzo[b,f]azepine-3-carboxylate* (**11a**). The oxime **9** (200 mg, 0.75 mmol) was dissolved in a mixture of CH_3_CN (5 mL) and DMF (1 mL). Triethyl amine (104 μL, 0.75 mmol) and methyl propiolate (67 μL, 0.75 mmol) were then added and the reaction was left at r.t. for 1 h under stirring. The reaction progress was monitored by TLC analysis (SiO_2_, AcOEt, *R_f_* **9** = 0.22, *R_f_* **11a** = 0.10). Acetonitrile was removed under reduced pressure and the DMF residue was dropped into water (20 mL). The brown precipitate was collected by filtration, washed with H_2_O and dried under vacuum to gave **11a** in a pure form (141 mg, 57%): ^1^H-NMR (DMSO-*d_6_*) δ 12.33 (s, 1H), 7.76–7.23 (m, 9H), 5.59 (br. s, 2H), 3.74 (s, 3H); ^13^C-NMR (DMSO-*d_6_*) δ 165.2, 156.7, 141.1, 140.1, 132.8, 131.9, 131.2, 130.0, 129.9, 129.5, 129.4, 128.4, 128.3, 128.2, 127.4, 127.3, 121.1, 113.4, 51.6; m.p. 198–199 °C; MS (CI^+^): *m/z* = 333 [MH]^+^; Anal. Calcd. for C_19_H_15_N_3_O_3_: C, 68.46; H, 4.54; N, 12.61; Found: C, 68.41; H, 4.63; N 12.49.

*3-(Phenylcarbonyl)dibenzo[b,f]pyrrolo[2,3-d]azepine-8(1H)-carboxamide* (**11b**). The oxime **9** (100 mg, 0.37 mmol) was dissolved in a mixture of CH_3_CN (2.5 mL) and DMF (0.5 mL). Triethyl amine (52 μL, 0.37 mmol) and phenyl propynone (49 mg, 0.37 mmol) were then added and the reaction was left at r.t. for 1 h under stirring. The reaction progress was monitored by TLC analysis (SiO_2_, AcOEt, *R_f_* **9** = 0.22, *R_f_* **11b** = 0.13). Acetonitrile was removed under reduced pressure and the DMF residue was dropped into water (10 mL). The yellow precipitate was collected by filtration, washed with H_2_O and dried under vacuum to gave **11b** in a pure form (101 mg, 71%): ^1^H-NMR (DMSO-*d_6_*) δ 12.41 (s, 1H), 7.82 (d, *J* = 7.4 Hz, 1H), 7.76–7.08 (m, 13H), 5.58 (br. s, 2H);^13^C-NMR (DMSO-*d_6_*) δ 191.1, 156.9, 141.0, 140.2, 139.8, 135.5, 133.4, 132.7, 131.5, 130.2, 130.1, 129.9, 129.5, 129.4, 129.1, 128.9, 128.8, 128.3, 128.2, 128.1, 127.6, 127.4, 122.7, 121.4; m.p. 265–266 °C; MS (CI^+^): *m/z* = 380 [MH]^+^, 337 [(M-HNCO)H]^+^; Anal. Calcd. for C_24_H_17_N_3_O_2_: C, 75.97; H, 4.52; N, 11.08; Found: C, 75.78; H, 4.55; N, 11.21.

*3-[(4-Nitrophenyl)carbonyl]dibenzo[b,f]pyrrolo[2,3-d]azepine-8(1H)-carboxamide* (**11c**). The oxime **9** (200 mg, 0.75 mmol) was dissolved in a mixture of CH_3_CN (4 mL) and DMF (1 mL). Triethylamine (104 μL, 0.75 mmol) and *p*-nitrophenyl propynone (131 mg, 0.75 mmol) were then added and the reaction was left at r.t. for 1 h under stirring. The reaction progress was monitored by TLC analysis (SiO_2_, AcOEt, *R_f_* **9** = 0.22, *R_f_* **11c** = 0.13). Acetonitrile was removed under reduced pressure and the DMF residue was dropped into water (20 mL). The brown precipitate was collected by filtration, washed with H_2_O and dried under vacuum to gave **11c** in a pure form (249 mg, 78%): ^1^H-NMR (DMSO-*d_6_*) δ 12.56 (br. s, 1H), 8.27–8.23 (m, 2H), 8.02–7.98 (m, 2H), 7.70–7.77 (m, 1H), 7.60 (s, 1H), 7.55–7.52 (m, 1H), 7.48–7.41 (m, 3H), 7.30–7.24 (m, 2H), 7.10 (dt, *J* = 7.6, 1.2 Hz, 1H) 5.63 (br. s, 2H); ^13^C-NMR (DMSO-*d_6_*) δ 189.1, 156.5, 149.4, 145.2, 140.8, 139.7, 132.7, 131.6, 131.2, 130.8, 130.4, 129.8, 129.3, 128.0, 127.9, 127.2, 127.1, 123.8, 121.7, 120.9; m.p. 188–189 °C; MS (CI^+^): *m/z* = 425 [MH]^+^, 382 [(M-HNCO)H]^+^; Anal. Calcd. for C_21_H_15_N_4_O_4_: C, 67.92; H, 3.80; N, 13.20; Found: C, 68.02; H, 3.93; N, 13.05.

*10-(Carbamoylhydrazono)-10,11-dihydro-5H-dibenzo[b,f]azepine-5-carboxamide* (**12**). The compound was prepared following the procedure present in the literature and the spectroscopic data were in accordance with those reported [8]. Yield = 85%.

*8H-[1,2,3]Thiadiazolo[4,5-d]dibenzo[b,f]azepine-8-carboxamide* (**13**). Thionyl chloride (5 mL) was cooled to −10 °C with an ice/NaCl bath, **12** (496 mg, 1.60 mmol) was slowly added and the reaction mixture was left rise to r.t. under stirring. The reaction progress was monitored by TLC analysis (SiO_2_, CH_2_Cl_2_/EtOH 95:5, *R_f_* **12** = 0.07, *R_f_* **13** = 0.35). When the reaction was complete (after about 2 h), the mixture was added drop wise to water (100 mL) and the pH was set to 7 adding an aqueous solution of NH_3_. The yellow precipitate was collected and dried under vacuum to afford **13** in a pure form (392 mg, 83%): ^1^H-NMR (CDCl_3_) δ 8.36 (d, *J* = 8.0 Hz, 1H), 7.72–7.59 (m, 6H), 7.46 (t, *J* = 7.5 Hz, 1H), 4.50 (br. s, 2H); ^13^C-NMR (CDCl_3_) δ 156.6, 155.9, 150, 140.6, 140.2, 132.4, 132.0, 131.5, 130.8, 130.5, 130.4, 129.5, 129.2, 129.2, 127.3; m.p. 231–232 °C; MS (CI^+^): *m/z* = 295 [MH]^+^, 323 [M+C_2_H_5_]^+^, 252 [(M-HNCO)H]^+^; Anal. Calcd. for C_15_H_10_N_4_OS: C, 61.21; H, 3.42; N, 19.04; Found: C, 61.28; H, 3.54; N, 19.11.

*8H-[1,2,3]Selenadiazolo[4,5-d]dibenzo[b,f]azepine-8-carboxamide* (**14**). **12** (470 mg, 1.52 mmol) was dissolved in acetic acid (5 mL) and SeO_2_ (202 mg, 1.82 mmol) was added portionwise while stirring. The temperature was rise to 118 °C and the mixture was left under stirring for 30 min. The reaction progress was monitored by TLC analysis (SiO_2_, CH_2_Cl_2_/EtOH 95:5, *R_f_* **12** = 0.07, *R_f_* **14** = 0.37). After cooling to r.t. water (100 mL) was added, the aqueous phase was extracted with ethyl acetate (3 × 40 mL), the combined organic layers were dried (Na_2_SO_4_) and the solvent was removed under reduced pressure to afford **14** as pink solid (250 mg, 48%): ^1^H-NMR (DMSO-*d_6_*) δ 8.15 (d, *J* = 7.7 Hz, 1H), 7.69–7.64 (m, 5H), 7.55 (m, 1H), 7.42 (m, 1H), 5.87 (br. s, 2H); ^13^C-NMR (DMSO-*d_6_*) δ 158.2, 156.7, 156.2, 142.1, 141.4, 141.4, 135.2, 132.8, 131.4, 131.1, 130.9, 130.4, 130.3, 129.3, 129.0; m.p. 233–234 °C; MS (CI^+^): *m/z* = 345 [M(^82^Se)H]^+^ (8.7%), 343 [M(^80^Se)H]^+^ (49.6%), 341 [M(^78^Se)H]^+^ (23.7%), 340 [M(^77^Se)H]^+^ (7.6%), 339 [M(^76^Se)H]^+^ (9.3%), 317 [(M(^82^Se)-N_2_)H]^+^, 315 [(M(^80^Se)-N_2_)H]^+^, 313 [(M(^78^Se)-N_2_)H]^+^, 312 [(M(^77^Se)-N_2_)H]^+^, 311 [(M(^76^Se)-N_2_)H]^+^; Anal. Calcd. for C_15_H_10_N_4_OSe: C, 52.80; H, 2.95; N, 16.42; Found: C, 52.74; H, 3.02; N, 16.26.

*10-Bromo-11-oxo-10,11-dihydro-5H-dibenzo[b,f]azepine-5-carboxamide* (**16**). The compound was prepared following the procedure present in the literature and the spectroscopic data were in accordance with those reported [26]. Yield = 92%.

*2-Amino-8H-dibenzo[b,f][1,3]thiazolo[4,5-d]azepine-8-carboxamide* (**17**). Compound **16** (300 mg, 0.91 mmol) and thiourea (205 mg, 2.69 mmol) were dissolved in ethanol (13 mL). The well stirred reaction mixture was brought to reflux for 3 h. The reaction progress was monitored by TLC analysis (SiO_2_, AcOEt/EtOH 9:1, *R_f_* **17** = 0.28). After cooling to r.t., the reaction mixture was dropped into water (100 mL), the white precipitate was collected and dried under vacuum to afford **17** in a pure form (120 mg, 43%). ^1^H-NMR (DMSO-*d_6_*) δ 7.78 (d, *J* = 7.5 Hz, 1H), 7.50–7.36 (m, 9H), 5.65 (br. s, 2H); ^13^C-NMR (DMSO-*d_6_*) δ 167.5, 157.0, 140.4, 139.9, 132.9, 131.4, 130.4, 130.3, 130.1, 129.1, 128.7, 128.6, 128.3, 120.6; m.p. 215–217 °C; MS (CI^+^): *m/z* = 309 [MH]^+^, 266 [(M-HNCO)H]^+^; Anal. Calcd. for C_16_H_12_N_4_OS: C, 62.32; H, 3.92; N, 18.17; Found: C, 62.43; H, 3.97; N, 17.98.

*2-Amino-8H-dibenzo[b,f][1,3]oxazolo[4,5-d]azepine-8-carboxamide* (**20**). **16** (200 mg, 0.60 mmol) and urea (181 mg, 3.02 mmol) were dissolved in *n*-butyl alcohol (3 mL), under a nitrogen atmosphere. The well stirred reaction mixture was brought to reflux for 16 h. The reaction progress was monitored by TLC analysis (SiO_2_, CH_2_Cl_2_/EtOH 95:5, *R_f_* **16** = 0.33, *R_f_* **20** = 0.03). After cooling to r.t., an aqueous solution of NaHCO_3_ was added to the reaction mixture and the solvent was removed under reduced pressure. Flash column chromatography (SiO_2_, CH_2_Cl_2_/EtOH 9:1, *R_f_* = 0.16) afforded **20** as white solid (56 mg, 32%): ^1^H-NMR (CDCl_3_) δ 7.80 (m, 1H), 7.54–7.33 (m, 7H), 5.79 (br. s, 2H), 5.04 (br. s, 2H); ^13^C-NMR (CDCl_3_) δ 161.1, 158.1, 137.0, 136.0, 130.9, 130.1, 129.8, 129.6, 129.0, 128.9, 128.6, 127.5, 127.0, 124.2; m.p. 224–226 °C; MS (CI^+^): *m/z* = 293 [MH]^+^, 250 [(M-HNCO)H]^+^; Anal. Calcd. for C_16_H_12_N_4_O_2_: C, 65.75; H, 4.14; N, 19.17; Found: C, 65.44; H, 4.11; N, 19.28.

*10H-Dibenzo[b,f]thiazolo[1',2':1,2]imidazo[4,5-d]azepine-10-carboxamide* (**21**). Compound **16** (200 mg, 0.60 mmol) and 2-aminothiazole (121 mg, 1.21 mmol) were dissolved in *n*-butyl alcohol (20 mL). The well stirred reaction mixture was brought to reflux for 6 h. The reaction progress was monitored by TLC analysis (SiO_2_, CH_2_Cl_2_/EtOH 95:5, *R_f_* **16** = 0.33, *R_f_* 2‑aminothiazole = 0.11, *R_f_* **21** = 0.06). After cooling to r.t., water was added to the reaction mixture and the solvent was removed under reduced pressure. Flash column chromatography (SiO_2_, CH_2_Cl_2_/EtOH 9:1, *R_f_* = 0.27) gave **21** as ochre solid (113 mg, 56%): ^1^H-NMR (DMSO-*d_6_*) δ 8.33 (d, *J* = 4.4 Hz, 1H), 7.92 (m, 1H), 7.82 (m, 1H), 7.61 (m, 1H), 7.56–7.45 (m, 7H), 5.62 (br. s, 2H); ^13^C-NMR (DMSO-*d_6_*) δ 169.8, 157.1, 151.6, 143.8, 139.9, 139.4, 132.9, 131.1, 130.2, 129.9, 129.5, 128.9, 128.7, 128.3, 128.2, 125.4, 120.3, 115.5; m.p. 257–258 °C; MS (CI^+^): *m/z* = 333 [MH]^+^, 290 [(M-HNCO)H]^+^; Anal. Calcd. for C_18_H_12_N_4_OS: C, 65.04; H, 3.64; N, 16.86; Found: C, 65.19; H, 3.59; N, 16.82.

*10H-Dibenzo[b,f]pyrido[1',2':1,2]imidazo[4,5-d]azepine-10-carboxamide* (**22**). Compound **16** (300 mg, 0.91 mmol) and 2-aminopyridine (128 mg, 1.36 mmol) were dissolved in *n*-butyl alcohol (30 mL). The well stirred reaction mixture was brought to reflux for 1 h. The reaction progress was monitored by TLC analysis (SiO_2_, CH_2_Cl_2_/EtOH 95:5, *R_f_* **16** = 0.33, *R_f_* 2-aminopyridine = 0.18, *R_f_* **22** = 0.06). After cooling to r.t., water was added to the reaction mixture and the solvent was removed under reduced pressure. Flash column chromatography (SiO_2_, CH_2_Cl_2_/EtOH 9:1, *R_f_* = 0.32) gave **22** as pink solid (157 mg, 53%): ^1^H-NMR (CDCl_3_) δ 8.71 (d, *J* = 7.0 Hz, 1H), 7.97–6.94 (m, 11H), 5.45 (br. s, 2H); ^13^C-NMR (DMSO-*d_6_*) δ 156.9, 146.4, 143.1, 141.9, 141.2, 135.4, 133.0, 131.1, 130.2, 129.9, 129.6, 129.0, 128.8, 128.5, 128.3, 126.9, 125.9, 125.5, 118.5, 112.9; m.p. 255–256 °C; MS (CI^+^): *m/z* = 327 [MH]^+^, 284 [(M-HNCO)H]^+^; Anal. Calcd. for C_20_H_14_N_4_O: C, 73.61; H, 4.32; N, 17.17; Found: C, 73.69; H, 4.28; N, 17.24.

*10H-Dibenzo[b,f]pyrimido[1',2':1,2]imidazo[4,5-d]azepine-10-carboxamide* (**23**). Compound **16** (100 mg, 0.30 mmol) and 2-aminopyrimidine (29 mg, 0.30 mmol) were dissolved in *n*-butyl alcohol (5 mL), under nitrogen atmosphere. The well stirred reaction mixture was brought to reflux for 8 h. The reaction progress was monitored by TLC analysis (SiO_2_, CH_2_Cl_2_/EtOH 8:2, *R_f_* **16** = 0.71, *R_f_* 2-amino-pyrimidine = 0.36, *R_f_* **23** = 0.28). After cooling to r.t., an aqueous solution of K_2_CO_3_ (0.30 mmol, 10 mL) was added to the reaction mixture and the solvent was removed under reduced pressure. Flash column chromatography (SiO_2_, CH_2_Cl_2_/EtOH 9:1, *R_f_* = 0.21) afforded **23** as white solid (33 mg, 34%): ^1^H-NMR (DMSO-*d_6_*) δ 9.29 (m, 1H), 8.72 (m, 1H), 8.06 (m, 1H), 7.97 (m, 1H), 7.69–7.49 (m, 6H), 7.44 (m, 1H), 5.69 (br. s, 2H); ^13^C-NMR (DMSO-*d_6_*) δ 152.5, 134.6, 131.2, 130.8, 130.2, 130.0, 129.2, 128.8, 128.7, 126.0, 110.5; m.p. 259–261 °C dec.; MS (CI^+^): *m/z* = 328 [MH]^+^, 285 [(M-HNCO)H]^+^; Anal. Calcd. for C_19_H_13_N_5_O: C, 69.71; H, 4.00; N, 21.39; Found: C, 69.60; H, 4.13; N, 21.33.

*5H-Dibenzo[b,f]azepine-10,11-dione* (**26**). Oxcarbazepine (**1**, 5.0 g, 19.82 mmol) and SeO_2_ (5.4 g, 48.67 mmol) in dioxane (100 mL) were brought to reflux under vigorous stirring for 7 h. The reaction progress was monitored by TLC analysis (SiO_2_, CH_2_Cl_2_/EtOH 95:5, *R_f_* **1** = 0.27, *R_f_* **26** = 0.56). After cooling to r.t. the orange precipitate was filtered and dried under vacuum to afford **26** (4.0 g, 90%): the spectroscopic data were in accordance with those reported in the literature [30].

*10H-Quinoxalino[2,3-d]dibenzo[b,f]azepine* (**27**). Compound **26** (100 mg, 0.45 mmol) and 1,2-diaminobenzene (49 mg, 0.45 mmol) in AcOH (2 mL) were refluxed for 1 h under stirring. The reaction progress was monitored by TLC analysis (SiO_2_, CH_2_Cl_2_/EtOH 95:5, *R_f_* **26** = 0.54, *R_f_* 1,2-diaminobenzene = 0.23, *R_f_* **27** = 0.69). The reaction mixture was left cooling to r.t. and added dropwise to stirred ice/water (25 mL), the yellow precipitate was collected by filtration, washed with water and dried under vacuum to afford **27** (64 mg, 49%): the spectroscopic data were in accordance with those reported in the literature [30].

*9H-Dibenzo[b,f]pyrazino[2,3-d]azepine-2,3-dicarbonitrile* (**28**). Compound **26** (100 mg, 0.45 mmol) and diaminomalonitrile (49 mg, 0.45 mmol) in AcOH (2 mL) were refluxed for 1 h under stirring. The reaction progress was monitored by TLC analysis (SiO_2_, CH_2_Cl_2_/EtOH 95:5, *R_f_* **26** = 0.54, *R_f_* **28** = 0.73). The reaction mixture was left cooling to r.t., the dark red precipitate was collected by filtration and dried under vacuum to afford **28** (86 mg, 65%): ^1^H-NMR (DMSO-*d_6_*) δ 8.13 (s, 1H), 7.70 (dd, *J* = 7.1, 1.4 Hz, 2H), 7.43 (td, *J* = 8.1, 1.5 Hz, 2H), 7.14 (t, *J* = 7.6 Hz, 2H), 7.11 (d, *J* = 8.1 Hz, 2H); ^13^C-NMR (DMSO-*d_6_*) δ 156.3, 153.6, 133.8, 132.6, 131.6, 126.7, 124.7, 121.4, 115.21; MS (CI^+^): *m/z* = 296 [MH]^+^; Anal. Calcd. for C_18_H_9_N_5_: C, 73.21; H, 3.07; N, 23.72; Found: C, 73.52; H, 3.04; N, 23.48.

*2,9-Dihydro-3H-dibenzo[b,f][1,2,4]triazino[5,6-d]azepin-3-one* (**29**). Semicarbazide hydrochloride (50 mg, 0.45 mmol) was dissolved in H_2_O (0.5 mL), this solution was added to a stirred solution of **26** (100 mg, 0.45) in AcOH (6 mL). The mixture was then refluxed for 1 h. The reaction progress was monitored by TLC analysis (SiO_2_, CH_2_Cl_2_/EtOH 95:5, *R_f_* **26** = 0.54, *R_f_* **29** = 0.25). When the reaction was complete, the solvent was removed under vacuum, water was added to the residue and the aqueous phase was extracted with AcOEt (3 × 10 mL). The combined organic layers were dried (Na_2_SO_4_) and the solvent was removed under reduced pressure. Flash column chromatography (SiO_2_, CH_2_Cl_2_/EtOH 95:5, *R_f_* = 0.25) gave **29** as red solid (23 mg, 19%): ^1^H-NMR (DMSO-*d_6_*) δ 8.23 (br. s, 1H), 7.89 (dd, *J* = 7.9, 1.6 Hz, 1H), 7.52 (dd, *J* = 7.8, 1.5 Hz, 1H), 7.45 (td, *J* = 7.8, 1.6 Hz, 1H), 7.33 (td, *J* = 7.6, 1.5 Hz, 1H), 7.17-7.14 (m, 2H), 7.10-7.04 (m, 2H), 5.75 (s, 1H);^13^C-NMR (DMSO-*d_6_*) δ 167.5, 153.9, 151.4, 148.4, 141.7, 134.2, 131.8, 131.1, 129.9, 125.2, 124.3, 123.7, 122.9, 120.8, 120.2; m.p. 227–230 °C dec.; MS (CI^+^): *m/z* = 263 [MH]^+^; Anal. Calcd. for C_15_H_10_N_4_O: C, 68.69; H, 3.84; N, 21.36; Found: C, 68.52; H, 3.95; N, 21.24.

*2-Phenyl-1,8-dihydrodibenzo[b,f]imidazo[4,5-d]azepine* (**30**). Compound **26** (300 mg, 1.34 mmol), benzaldehyde (143 mg, 1.34 mmol) and ammonium acetate (268 mg, 3.48 mmol) were dissolved in AcOH (12 mL). The reaction mixture was brought to 80 °C for 1 h under stirring. The reaction progress was monitored by TLC analysis (SiO_2_, CH_2_Cl_2_/EtOH 95:5, *R_f_* **26** = 0.54, *R_f_* **30** = 0.28). The mixture was cooled to r.t and dropped into ice/water (50 mL). The aqueous suspension was extracted with AcOEt (3 × 20 mL) and the unreacted diketone was removed by filtration. The organic phase was dried (Na_2_SO_4_) and the solvent was removed under reduced pressure. Flash column chromatography (SiO_2_, CH_2_Cl_2_/EtOH 95:5, *R_f_* = 0.28) gave **30** in a pure form (115 mg, 28%): ^1^H-NMR (DMSO-*d_6_*) δ 12.38 (br. s, 1H), 8.09 (m, 2H), 7.62 (m, 1H), 7.49 (m, 2H), 7.41 (m, 2H), 7.22 (s, 1H), 7.06 (m, 2H), 6.94–6.84 (m, 4H);^13^C-NMR (DMSO-*d_6_*) δ 148.3, 147.9, 147.8, 139.2, 131.1, 129.9, 129.6, 129.5, 129.5, 129.3, 129.1, 127.7, 127.3, 126.7, 126.2, 126.2, 123.2, 123.0, 122.9, 120.8, 120.5; m.p. 102–103 °C; MS (CI^+^): *m/z* = 310 [MH]^+^; Anal. Calcd. for C_21_H_15_N_3_: C, 81.53; H, 4.89; N, 13.58; Found: C, 81.58; H, 4.95; N, 13.46.

*9H-Dibenzo[b,f]pyrido[1',2':1,5]pyrazolo[3,4-d]azepine-9-carboxamide* (**32**). Oxcarbazepine (**1**, 300 mg, 1.19 mmol), 1-aminopyridinium iodide (317 mg, 1.43 mmol) and potassium carbonate (493 mg, 3.57 mmol) were dissolved in a mixture of H_2_O (20 mL) and *i*-PrOH (20 mL). The temperature was rise to 100 °C and the mixture was left under stirring for 2 h. The reaction progress was monitored by TLC analysis (SiO_2_, CH_2_Cl_2_/EtOH 95:5, *R_f_* **1** = 0.27, *R_f_* **32** = 0.18). Isopropanol was removed under reduced pressure and the aqueous residue was extracted with ethyl acetate (3 × 20 mL). The combined organic layers were dried (Na_2_SO_4_) and the solvent removed in vacuum. Flash column chromatography (SiO_2_, CH_2_Cl_2_/EtOH 9:1, *R_f_* = 0.36) gave **32** as red solid (85 mg, 22%): ^1^H-NMR (DMSO-*d_6_*) δ 8.91 (d, *J* = 6.9 Hz, 1H), 8.07 (d, *J* = 9.0 Hz, 1H), 7.94 (m, 1H), 7.83 (m, 1H), 7.61–7.40 (m, 7H), 7.12 (t, *J* = 6.8 Hz, 1H), 5.60 (br. s, 2H); ^13^C-NMR (DMSO-*d_6_*) δ 157.1, 149.9, 142.7, 141.2, 138.0, 131.6, 131.5, 130.9, 130.7, 130.5, 130.2, 129.1, 128.9, 128.7, 128.4, 128.2, 126.3, 118.3, 114.7, 108.6; m.p. 254–255 °C; MS (CI^+^): *m/z* = 328 [MH]^+^, 284 [(M-HNCO)H]^+^; Anal. Calcd. for C_20_H_14_N_4_O: C, 73.61; H, 4.32; N, 17.17; Found: C, 73.49; H, 4.43; N, 17.10.

## 4. Conclusions

To summarize, a structurally diverse heterocyclic library sharing a common dibenzo[*b,f*]azepine scaffold, decorated with [*d*]-fused heterocycles has been reported, thus providing a foundation for the further investigation of their biological activities.
